# Palliative and End-of-Life Care Access for Immigrants Living in High-income Countries: A Scoping Review

**DOI:** 10.1177/23337214231213172

**Published:** 2023-11-22

**Authors:** Gertrude Gondwe Phiri, Joyce Muge-Sugutt, Davina Porock

**Affiliations:** 1 Edith Cowan University, Joondalup, WA, Australia

**Keywords:** palliative care, decision-making, end-of-life, advance care planning

## Abstract

This scoping review aimed to explore what is known about palliative and End-of-Life (EOL) care access by immigrants with culturally and linguistically diverse (CALD) background living in high-income Organization for Economic Co-operation and Development (OECD) countries. CaLD immigrants have low utilization of palliative care services with patients’ family members taking up the role of caring, leading to immigrants not fully benefiting from the specialized services that are offered to alleviate suffering and promote quality of life. While there is some research in this area mainly in Europe, it cannot be said about all high-income OECD countries. Achieving person-centered care in high-income countries, requires identifying and addressing barriers to care access, especially by immigrants with CaLD background. Five-stage methodological framework by Arksey and O’Malley was used to undertake the review. Immigrants in OECD countries experience challenges in accessing palliative and EOL care services. The review also identified limited literature on the subject and establishes need for more research on the subject.

**What this paper adds**

1.  Challenges health care professionals to establish at the onset, EOL preferences of patients from CaLD backgrounds especially in the area of decision-making.
2.  Health care professionals and patients to co-designs EOL care strategies.
3.  Health professionals to consider incorporating spiritual care and or other treatments that maybe unfamiliar in the healthcare setting but can bring comfort to the patient.

Applications of study findings

1.  At policy level; as policy makers consider improvements and reviews to palliative care for CaLD populations in the local geographical areas.
2.  At healthcare professional level; training staff on how to approach the subject with patients who consider it taboo.
3.  At CaLD community level; sensitization of the community to work with healthcare workers, to feel comfortable to express what their care needs are so that care strategies can be co-designed.

## Introduction

Caring for individuals during palliative and EOL stage, reflects compassion as symptoms are managed, to promote comfort for the patient (Department of Health, 2018). Immigrants from CaLD background in high-income OECD countries have low utilization of palliative and EOL care services. As a result, patients’ family members take up the role of caring, leading to immigrants not fully benefiting from the specialized services that are offered, to alleviate suffering and promote quality of life (Shabnam et al., 2022). The situation has become more pronounced due to the increased rates of movement of people across boarders from low to high-income countries, the final destination for most immigrants (McAuliffe et al., 2022). This has created culturally diverse communities, posing challenges in attaining the desired quality palliative and EOL care for all, especially CaLD immigrants (World Palliative Care Alliance, 2014).

Globalization, the interdependence of economies, cultures, populations, and other human activities across borders, is a concept that dates back centuries. However, it became more prolific in the early 1960s with easier and quicker travel (d’Aiglepierre et al., 2020; Triandafyllidou, 2018). Technology has also played a big part in globalization, facilitating exchange of information and migration. Inevitably, this has led to migrants dying away from their country of origin (Bray et al., 2018; OECD, 2017). Literature reviews undertaken in this area in the recent past identified communication, culture (Gerber et al., 2020; Kwok et al., 2020; Shabnam et al., 2022), limited knowledge and awareness of health information (Gerber et al., 2020; Shabnam et al., 2022), collectivism (Gerber et al., 2020; Kwok et al., 2020) and lack of focus from migrants’ perspective (Kwok et al., 2020) as barriers to utilization of palliative care services by immigrants.

Worldwide, there were 272 million international migrants in 2019. Of the total migrant population, 120 million (over 44%) were to OECD countries (d’Aiglepierre et al., 2020; OECD, 2022), because of economic benefits (OECD, 2022; Phillips & Spicks, 2012). The OECD is an organization of 38 market-based economies which work collaboratively to develop policy and standards for sustainable economic development (Woodward, 2009). Within the OECD, there are low-income countries but, migrants target the high-income countries as their destination. This migration trend is projected to continue; therefore, it is important to incorporate practices that would meet person-centered palliative and EOL care needs of all, including immigrant populations.

A scoping review of relevant articles published between 2009 and 2021, was conducted to explore palliative and EOL care access by CaLD immigrants in high income countries. Included in the review were papers from Australia, Canada, Sweden, New Zealand, and the United States of America.

## Aim of the Review

The aim of the scoping review was to gain greater understanding of access to palliative and EOL care services by CaLD immigrants in high income countries.

## Review Question

What is known about CaLD immigrants in high-income countries access to palliative and EOL care services?

## Methods

The scoping review utilized the five-stage methodological framework namely, identifying the research question, identification of relevant studies, study selection, charting of data, and collating and presenting the results (Arksey & O'Malley, 2005).

## Inclusion and Exclusion Criteria

Pre-planned inclusion and exclusion criteria were used to maintain consistency. The first author conducted the review with the oversight of co-authors who are also supervisors, to ensure relevance, accuracy, and high quality of evidence available. Literature included were of primary research studies from 2009 to 2021, from high-income countries of the OECD, related to palliative and end-of-life care access by CaLD immigrants, with full text and in English. Excluded were literature over the same period on palliative and EOL care access by indigenous or minority ethnic groups. Articles about palliative care for immigrants of English-speaking background were not part of the review. Also excluded were articles from low to medium-income countries of the OECD, as well as systematic and scoping reviews. Reference lists were reviewed for additional resources.

## Search Strategy

A comprehensive strategy was developed for literature search by the authors which included the search terms “Palliative care,” “end-of-life care,” “cultur*,” “migrant*,” and “OECD countries.”

The literature search was conducted in November 2022 using Google Scholar, Medline, CINAHL, and PsychINFO databases.

## Data Charting

Data analysis was facilitated by extraction of data from included articles which were recorded onto a spreadsheet under the following headings: Author/s and Year Published, Study aim/s, Sample and Methods, Where study conducted, and Key findings (Peters et al., 2020). Data extraction (Table 1) Appendix 1, provides information about articles included in the scoping review.

## Selection of Evidence

Articles reviewed included two New Zealand qualitative and interpretive phenomenology studies (18 and 10 participants respectively), five Australian studies using survey (239 participants), case study (1 participant), focus group (15 participants), qualitative descriptive study (a family of six), and a qualitative study (30 participants). Other articles included were a Swedish study using national quality register to investigate 81,418 deceased patients in relation to palliative care, a Canadian focus group study (6 participants) and two United States of America (USA) studies; a case study (1 participant) and a qualitative study (13 participants).

## Findings

**Figure 1.**
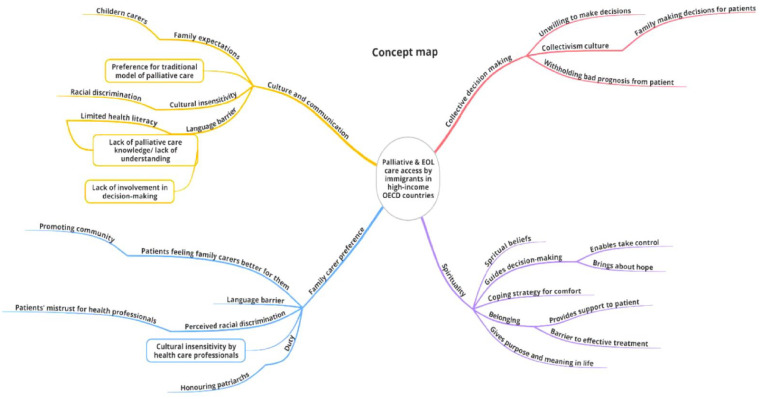
Palliative and EOL access concept map.

This literature revealed barriers to accessing palliative and EOL care by immigrants in high-income OECD countries. The identified barriers are discussed under four main themes including: culture and communication; collective decision making; spirituality; and family carer preference. Figure 1 illustrates the complexities related to palliative and EOL care access by immigrants of CaLD background.

Figure 1 illustrates the four co-concepts of barriers to accessing palliative and EOL care by immigrants of CaLD background in OECD countries. Each co-concept commences with elements in the peripheral, which culminate in the identified co-concepts largely responsible for negatively impacting the care service access by immigrants of CaLD background.

## Culture and Communication Barriers

All the studies discussed culture, two of them specifically included culture and communication barriers (Hiruy & Mwanri, 2014; Kirby et al., 2018). Culture plays an important role in how individuals understand, experience, and manage illness (Germov, 2014) including palliative and EOL care choices and decision-making. The literature suggests that lack of access to, and use of palliative and EOL care services by the CaLD immigrant population in high-income countries, was partly due to cultural and communication barriers (Bray et al., 2018; Hiruy & Mwanri, 2014). Knowledge about palliative and EOL care services was minimal or absent in these communities and this also was attributed to communication and cultural barriers (Sneesby et al., 2011). For example, participants reported that in Sudan, they relied on herbs and plants for medicinal purposes which were not available in Australia. The palliative care concept was described to participants by the researcher, as care aimed at promoting and improving quality-of-life for the dying person by managing symptoms. After gaining an understanding of the concept, older community members reported a preference of the traditional model of families taking care of the dying without involvement of specialist palliative care teams, while the younger members of the community were observed to be receptive of the western palliative care concept. Older community members sought medical treatment for symptoms however, participants reported that the prescribed treatment may either be discontinued or medications may be shared with other family members, rendering treatment ineffective (Sneesby et al., 2011).

In another study, older adults and adult children’s groups preferred family carer arrangements, citing language barriers and cultural insensitivity by healthcare workers (Eckemoff et al., 2018). For participants who migrated to the USA as adults, language was a significant barrier as most participants did not speak English and there was no palliative care information written in Russian accessible to them. Hospice staff also alluded to the fact that it was a challenge to care for clients from a different linguistic and cultural background due to lack of knowledge about patients’ cultures and language. Similar cultural issues were also identified with Latino patients in the USA (Smith et al., 2009).

In their study, Smith et al. (2009) explored ways of improving care delivery to immigrants in the USA. The participant expressed culture and racial discrimination as some of the barriers to accessing palliative care. In addition to the perception of cultural and racial discrimination, this participant had health literacy challenges which resulted in communication barriers. As a result, relied on family for explanation of the health information provided by health care staff. This usually led to patient’s lack of understanding of information provided as well as lack of involvement in decision making.

The two USA studies (Eckemoff et al., 2018; Smith et al., 2009) found that cultural barriers and racial discrimination led to mistrust for the clinical care system. The hospice staff confirmed that the care provided to CaLD was not up to standard, stating that communication with patients was the main reason for providing “substandard” care (Smith et al., 2009). Culture as a barrier to accessing palliative care was not unique to studies in the USA, New Zealand reported similar findings.

Bray and Goodyear-Smith (2013) study also reported that culture was one of the major barriers to accessing palliative care. The findings were again observed in Bray et al.’s (2018) phenomenological study. Participants preferred to receive care at home to promote continuity of social connection to their community. Family caregivers, predominantly adult children of patients, wanted to provide EOL care in their own homes as a way of honoring the patriarchs which was very important in their culture (Bray et al., 2018). The similarity in the findings suggests that not much, if at all, had changed in 5 year between the two studies.

The theme of culture influencing palliative and end-of-life care resonates through many studies of immigrant populations. For instance, the study of the terminally ill among Indian immigrants in Australia (Shanmugasundaram & O'Connor, 2009) also identified cultural insensitivity as a barrier to accessing formal palliative and EOL car. Families reported that they preferred to care for their dying relatives within the family. Additionally, the study also found that caring for the dying by family members in this immigrant community, was perceived as an honor by the family carer, similar to Bray et al.’s (2018) study findings. Australian studies have also reported communication as barrier to accessing palliative and EOL care by immigrants adding that linguistic difficulties contributed a great deal to care access. It is important to understand patients’ cultures in order to communicate in a manner which will not be construed as insensitive, bearing in mind that in some cultures, discussing palliative and EOL, including death, can be seen as taboo (Gerber et al., 2020).

The Canadian study on awareness of palliative care and EOL opioids use (Maddalena et al., 2013), had similar findings as the Australian study among the Sudanese, and included the issue of not being aware of available palliative and EOL care services (Hiruy & Mwanri, 2014). These studies demonstrate that communication can be a barrier to health literacy in relation to palliative and EOL care which can lead to limited access to services. While the studies identified above found communication and culture as barriers to accessing palliative and EOL care, one study (Carlsson & Hjelm, 2021) only reported on the difference in access to palliative care between Swedish-born patients and foreign-born, reporting that the Swedish-born patients accessed the services more than the foreign-born. The reason for not providing more information on the access ratios between the two population was because the study was conducted using deceased patients’ registers.

## Collective Decision Making

Collectivism is a cultural phenomenon where people within the group are interdependent and prioritize the objectives of the group over individual goals (Krassner et al., 2017). Collectivist decision-making is predominantly practiced in simple, tight indigenous communities (Triandis & Suh, 2002) whose values are focused on the wellbeing of the extended networks not just immediate family members (Krassner et al., 2017). There are two types of collectivism identified in literature; vertical which emphasizes on solidarity and respect for authority within the group and, horizontal which values empathy, sociability and cooperation in the community (Triandis & Gelfand, 1998). Communities that adhere to collectivism values usually practice the latter as demonstrated in three studies included in this review (Hiruy & Mwanri, 2014; Jeong et al., 2015; Sneesby et al., 2011). In these communities, important decisions such as accepting that the individual is dying therefore, not requiring life-saving treatment, are generally made at family or community level (Hiruy & Mwanri, 2014).

To illustrate the horizontal collectivist approach, Hiruy and Mwanri (2014) reported that the individual at the center of the event may participate in the discussion in relation to their goals of care but, final decisions were made by elders in the family or community group. Family could withhold information provided by the health care professionals if they felt that the information would be too big a burden to bear by the person (Hiruy & Mwanri, 2014; Yoong, 2015). Sneesby et al. (2011) in their study with African immigrants in Australia, found that bad news about disease prognosis was provided to the patient only when it was felt that the patient had adequate support to handle and process the news, thereby preventing any potential self-harm. Smith et al.’s (2009) also identified collectivist decision-making practices as decisions were made by the patient’s partner without involvement of the patient. The authors reported that the patient had a different opinion about her care from her partner who was unwilling to talk about comfort measures. His focus was on treatment and cure, even after being advised that the leukemia was very aggressive and nothing else could be done (Smith et al., 2009).

Collectivist cultures as opposed to individualism (Hiruy & Mwanri, 2014; Jansky et al., 2019) could be the reason why decision-making is considered a communal responsibility in some communities. The Australian study about awareness of advance care planning in older patients of CaLD background demonstrated the collective decision-making practice and found that advance care planning uptake was low (Jeong et al., 2015). This could suggest unwillingness by patients to make EOL decisions, leaving that to their children to decide on their behalf, reinforcing the collectivist values. Considering Smith et al. (2009) example, staff, as patients’ advocates have an obligation to check with patients if they want to make their own decisions or would like to rely on the family. In addition to communication, spirituality also plays a significant role in seeking quality palliative and EOL care.

## Spirituality

The theme of spirituality was identified in four studies included in this review (Hiruy & Mwanri, 2014; Kirby et al., 2018; Smith et al., 2009; Sneesby et al., 2011). Spirituality is considered an important part of an individual, whether they are religious or not (Shaw et al., 2016). Coping during times of ill-health can be difficult and spirituality has been reported to be a source of comfort for some immigrant CaLD communities (Hiruy & Mwanri, 2014; Kirby et al., 2018; Sneesby et al., 2011). Hiruy and Mwanri (2014) observed in their study that, spirituality was an important aspect during EOL care for the patient. Decisions made during this time, were based on spiritual beliefs and cultural norms. In addition, religious leaders took a leading role in visiting and encouraging the patient as he went through a difficult time. The community to which the patient belonged was religious too and, was also a source of comfort for the patient. Another Australian study, demonstrated religious faith can be a coping mechanism when one is faced with life threatening situations (Kirby et al., 2018).

In addition, spirituality is said to give a person purpose in life and guidance in decision making when faced with dilemmas in life (Cassidy & Davies, 2006; Matandiko, 1996; Shaw et al., 2016). This aligns with what psychologists have identified in people faced with difficult situations in health matters (Shaw et al., 2016). Cassidy and Davies (2006) also assert that, apart from giving meaning and purpose, spirituality may help individuals identify their role in their EOL care and in turn, enable them take control of situations, bringing about hope. Having hope through spirituality when in a dire situation can be seen in Smith et al. (2009) study.

Smith et al.’s (2009) demonstrated that spirituality could give hope when the situation is irretrievable. However, what is perceived as a coping strategy could also be a barrier to effective symptom management in palliative and EOL care as this study demonstrated. The patient’s need for comfort measures was ignored because the partner who was the decision maker, was focused on praying for her to get better, insisting on medical staff to persist with treatment. Ineffective symptom management may lead patients to assume that being cared for by family, would be a better option.

## Preference for Family Carer Over Healthcare Professional

Cultural and communication barriers as well as collective decision making, are precursors to individuals preferring to be cared for at home and families wanting to care for their loved ones (Eckemoff et al., 2018; Shanmugasundaram & O'Connor, 2009). While some acknowledge that assistance from EOL care service provider organizations would be the best way to care for their loved ones at home, most immigrants feel it is their obligation and honor to provide EOL care to their family member (Eckemoff et al., 2018; Shanmugasundaram & O'Connor, 2009). This is perceived as a duty they must do, and the patient feels that they must be cared for by family. Others choose to be cared for by family because of distrust of health care workers due to poor adaptation to the host country (Jansky et al., 2019).

Older Russians preferred family to look after them and their children preferred to be carers, stating that they would not wish to have their parents “locked up.” However, in the same study, there were other participants who expressed different opinion, stating that the society needed to take some responsibility in caring for seniors (Eckemoff et al., 2018). This was a single voice amongst many, as most older immigrants felt caring for parents on EOL was the responsibility of their children.

## Discussion

There has been a call within these countries to address immigrants’ access to palliative and EOL care services yet, there is still a long way to go to realizing the dream of accessible palliative care for all. While accurate estimates of how many immigrants utilize the services are not available, it is evident from the literature reviewed that there are significant barriers that need to be urgently addressed to turn the tide.

Experiencing EOL free from pain and with dignity, is something that is widely accepted however, the manner in which these are achieved vary from one community to another, largely dependent on the culture (Clark, 2012; Green et al., 2019; Speck, 2016). Culture is critical in an individual’s life; significant decisions are shaped by one’s cultural beliefs. When individuals emigrate, they carry with them their cultures which mostly are different from that of the host country. To access care by immigrants with CaLD background, there needs to be an understanding of, and respect for their cultural needs by the palliative and EOL care service providers (Green et al., 2019). Historically, discrimination, racial profiling, and marginalization are realities that impact on seeking health care services (Schuster-Wallace et al., 2022). The solution lies in addressing the identified vices so that immigrant communities can develop trust in the health care system, something that has been eroded. Establishing groups to engage with CaLD immigrant communities in OECD countries on issues of health, would be of benefit to both care providers and service users (Quinn & Hickey, 2008). Such an engagement would assist in establishing beliefs, values, and practices of CaLD communities which do not always correspond with palliative care policies. Once patterns are established, they would serve as a guide for policy reviews in seeking to match them according to patients’ needs (Clark, 2012) with involvement of service users (Sbaiti et al., 2021).

Cultural sensitivity by health care providers toward CaLD patients, is of paramount importance in encouraging them to utilize the services especially EOL care. Sensitivity requires recognition that not all cultures are the same, or even similar and that an effort should be made in identifying the different cultures of patients to gain confidence and trust (Schuster-Wallace et al., 2022). Culture specific engagement in palliative care is the key to accessibility by immigrant communities (Quinn & Hickey, 2008).

Cultural context and effective communication can promote comfort for the patient, essential for making informed decisions by patients in the palliative care situation. Therefore, it is important for service providers to be competent in culturally effective communication (Green et al., 2019; Long, 2011). Communication is an important part of interaction required between service users and service providers. Literacy deficits, compounded by being in a new environment, with an unfamiliar health care system can be quite daunting for immigrants. Older immigrants usually tend to depend on their children for interpretation when they visit the health care system (Sungur et al., 2022). However, attending primary health care with a family member may not always be possible. Setting up communication tools such as language cards at general practitioners, the first point of contact for immigrants in the health care system, would assist in providing information, and promoting appetite to access health care services by CaLD communities (Saito et al., 2021). General practice needs to be equipped with knowledge, skills, and time to enable them to orient the new immigrants to the new health care system. This would enable immigrants to be confident in the system which would translate into usage of the health care services. Good and honest communication is foundation to accurately assessing patients’ needs and communicating empathy thereby promoting comfort (Amoah, 2011). Communication is all encompassing, however, non-verbal which is related to body expression, can be powerful where language barriers exist (Richmond & McCroskey, 2019; Steinfatt & Millette, 2019). Body language can transmit either positive or negative messages to the patients which can promote trust or reinforce the already existing barriers. Service providers must therefore be aware of their own communication skills and be able to read the non-verbal communication from their patients (Byrne, 2008). Communication also plays vital role in helping patients understand information provided relevant for making decisions.

## Collective Decision-Making

Differences in decision-making process between CaLD immigrants and health care providers translate into differences in the understanding of the issue with the two groups having different expectations (Cain et al., 2018). Palliative care is based on individualistic approach and there is an expectation that the patient themselves get a say in their care. In CaLD communities, families are usually intermediaries between patients and service providers, shifting the autonomy that western medical systems emphasize on, leading to discomfort on the side of care providers (Bullock, 2011; Ho et al., 2010). This is a clash that creates a problem and results in tension and the palliative care team maybe getting frustrated with that form of decision-making process. To address that tension, palliative care professionals, need to be bolder in asking about how the patient wants to make decisions, who they want involved in decision-making, how to disseminate any information to the patient, if they would like to receive the information themselves or through significant others. Allowing the patient to nominate how they would like important issues in care partnership addressed, would amount to a true person-centered care approach, unlike expecting the patient to conform to the palliative care team’s expectations. To reach that level of conversation with the patients, care providers must approach the role with an open mind, with respect to their patients, and acceptance of the decision-making process that the patients choose. Immigrants must, therefore, be given the opportunity on how much they want to be involved but also, respected if they decide to have less involvement in the decision-making process. Additionally, the preference of who cares for them must also be respected, a decision that is usually made along the lines of spirituality.

Like culture, spirituality plays a significant role when individuals are faced with something that requires a major decision to be made (Amoah, 2011; Byrne, 2008). It is at the core of an individual and this aspect of the person must be understood by those providing care (Speck, 2016). Spirituality is as important as psychological, social, and physical health in its contribution to improving quality of life to those experiencing life threatening illness. However, health professionals usually relegate it to the background (Amoah, 2011). Relegating spirituality to the background is in contrast with the literature which suggests that spirituality provides meaning to life, helps one find identity and, acts as a coping mechanism (Byrne, 2008; Cassidy & Davies, 2006). Since spirituality is so vital in how an individual copes with difficult situations, it would be of benefit to concurrently incorporate spiritual activities and use of conventional treatment for symptom management to promote comfort for patients. Coming to an agreement on how both spiritual activities, like praying, and administering conventional treatment for symptom management would be applied side-by-side without causing conflict would be of benefit for both the patient and caring team.

Individuals have varied beliefs which may not align with those of the caring team members. It is important to first acknowledge own spirituality position, be comfortable with that position then come to the caring role with the understanding that those in their care, could have different spiritual beliefs (Amoah, 2011).

Holistic care can be provided when all the elements of a whole person are considered because even though one maybe facing death, spirituality provides belonging, self-worth, and faith which are transcendent, and provides hope (Byrne, 2008). The hope that comes with spirituality guides individuals in decision-making including choosing to be cared for by family members.

## Family Carer Preference

Treatment practices and traditional models of caring for dying family members is considered a family responsibility and an honorable act by the carer in CaLD communities (Eckemoff et al., 2018; Shanmugasundaram & O'Connor, 2009). Palliative and EOL care services aim to honor that, by partnering with families, to care for their loved ones (Fahlberg et al., 2016). This model is not well understood by a large proportion of the immigrant community who believe that seeking palliative care services is equivalent to abandoning their family member in need (Shanmugasundaram & O'Connor, 2009). This view demonstrates that people have different ideas of what palliative care approach is all about, and not understanding what is meant by the patient and family being the unit of care (Alam et al., 2020). These extreme views can also be noted where children of palliative care patients felt that having their parents managed by palliative care professionals, was like locking them up (Eckemoff et al., 2018). The rationale for such perceptions could be because, when faced with difficult situations, people retreat to their comfort zone (Germov, 2014; Kolcaba, 2003) which in this case, may be compounded by being in a new country. If palliative care services are to be utilized by immigrant communities in high-income OECD countries, the issues identified above need to be addressed.

Although barriers to palliative care are well documented (Nyatanga, 2002; Schuster-Wallace et al., 2022), much still needs to be done. Literature continues to emphasize on the need to raise awareness and have education for both palliative care professionals and the different immigrant groups. Awareness and education are essential for ensuring that accurate and beneficial information is disseminated to the targeted populations. This review provides pointers to what needs to be addressed for palliative and EOL care access by immigrants in OECD countries to gain momentum. There must be a different approach in the way the barriers are addressed, if a change is to be realized.

## Conclusion

This study has provided valuable insight into issues of limited access to palliative and EOL care services by immigrants in OECD high-income countries. In this review, 11 articles were included in the analysis, from the palliative and EOL care access by immigrants in high-income OECD countries perspective. The literature reviewed demonstrated that culture and communication were barriers to access to care by the immigrant communities. Additionally, spirituality, and collective decision-making, were also identified as points of tension between palliative care patients and professionals. This paper advances that palliative care professionals be bolder to ask patients about preferred channels of communication, how or, who would be making decisions for the patient and how much of the care process the patient would like to be involved in. If the patient preferred not to be involved, their preference must be respected even when it is strongly preferred by professionals that the patient participate in their care delivery.

## Figures and Tables

**Figure 2.**
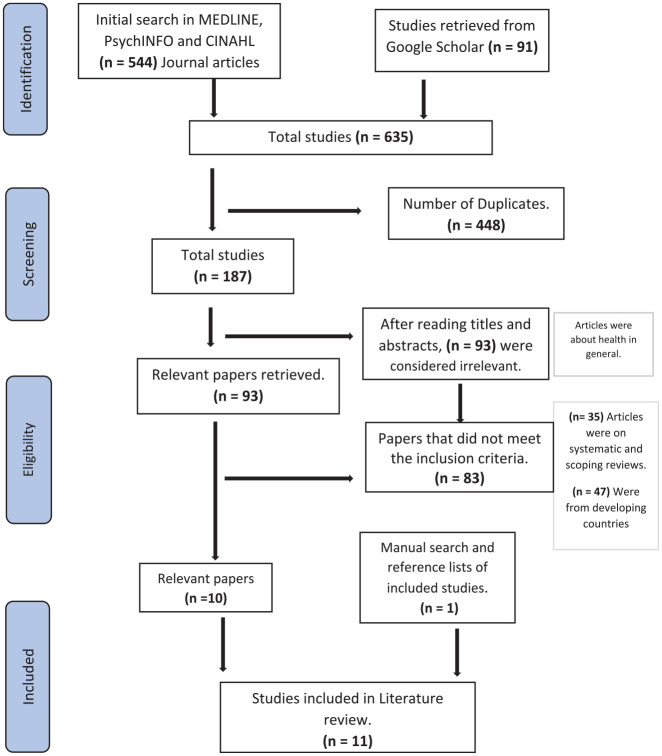
Prisma flow chart (Briggs, 2020 ).

## Appendix 1

**Table 1.** Data Extraction Table.

	List of reviewed studies (11 in total)					
	Author/s & year	Study	Study aim/s	Sample and methods	Where conducted	Results
1	Bray and Goodyear-Smith (2013)	Patient and family perceptions of hospice services: “I knew they weren’t like hospitals”	To gain a patient-and-their-family perspective on the hospice, including exploration of components of service care that could be improved for various cultural groups.	18 palliative care patients or carer family members, ranging in age from 39 to 81 years, who reflected the ethnic diversity of the population of the region using qualitative study technique.	New Zealand	Key themes emerged – 1. hospice personnel’s approach to patients, 2. quality of service, 3. cultural barriers, and 4. strategies for future improvement.
2	Bray et al. (2018)	Exploring the lived experience of migrants dying away from their country of origin.	To explore the lived experiences of migrants dying away from their country of birth or origin	10 participants, migrants to New Zealand, recruited through four hospices. Semi-structured interviews were used in Interpretive Phenomenology.	New Zealand	1. Living with 2 identities bringing about a feeling of aloneness, while for some that brought about a sense of belonging. Social connection was important. 2. Migration brought about thoughts of having attained a better life, but lost dreams because of the illness and also reminiscing about their country of origin. 3. Making immediate plans of what needs to be accomplished before dying.
3	Jeong et al. (2015)	“Planning ahead” among community-dwelling older people from culturally and linguistically diverse background: a cross-sectional survey.	To explore preparedness of end-of-life care planning among community-dwelling older persons of culturally and linguistically diverse background.	229 community older adults (65+) who attended 17 day care centres using survey study.	Australia	Awareness of advance care planning was low, and completion of advance care directive was very low. 37.5% of Anglo Celtic group had an enduring guardian, compared with 15.5%, 24.1%, and 13.3% from Mediterranean, Eastern European and Asia/Pacific group, respectively. Children were the most preferred substitute decision-makers more for Asia/Pacific group than Anglo Celtic, Mediterranean and Eastern Europeans. The various difficulties experienced included being time-consuming, difficult to understand terms and forms, and do not know how to do it.
4	Hiruy and Mwanri 2014	End-of-life experiences and expectations of Africans in Australia: cultural implications for palliative and hospice care.	To explore sociocultural end-of-life experiences of Africans and their interaction with the health services.	One Sudanese male participant of refugee background, using case study.	Australia	1. Culture plays a significant role in end of life for an African migrant. 2. Decision making in important matters such as end of life, is a community responsibility. 3. Religion and religious leaders play a vital role in end of life matters in the Sudanese community. 4. It is very important for immediate and extended family to visit the sick before they die.
5	Sneesby et al. (2011)	Death and dying in Australia: perceptions of a Sudanese community.	To obtain information to support Palliative Care health workers to meet the needs of Sudanese population in death, dying and bereavement.	15 participants were recruited. Four focus group interviews were conducted. Each focus group comprised of three to four participants.	Australia	1. Participants were not aware of the palliative care concept initially. 2. Older members of the Sudanese community like the traditional concept of family caring for the sick/dying while the young ones prefer the western approach. 3. Consensus that bad news was withheld from patients and family until support was available. 4. Faith is an important factor for Sundanese—Christianity or Islam.
6	Shanmugasundaram and O'Connor (2009)	Palliative care services for Indian migrants in Australia: experiences of the family of terminally ill patients.	To explore the issues related to accessing palliative care services for Indian migrants, identify the effectiveness of palliative care in supporting the patient and family and to recommend strategies for improving this care.	Participants were six, members of one family. In-depth interviews were used in a qualitative descriptive design.	Australia	1. Challenges in accessing care. Lack of sensitivity by health staff was identified as the reason. 2. Cultural issues; food was a factor; described as medicine. 3. Caring for a dying family member is seen as an honour. 4. Presence of large numbers of family member as death draws near.
7	Maddalena et al. (2013)	Awareness of Palliative Care and End-of-Life Options Among African Canadians in Nova Scotia.	To assess the knowledge African Canadians living in Nova Scotia have regarding their options for palliative and end-of-life (EOL) care.	Six caregivers from the black communities who had looked after someone who died. Focus group interviews were utilised.	Canada	1. There was a cultural expectation that family/community members would provide the bulk of the care required at home. 2. Caregivers had very little information about accessing palliative care services. 3. Health system lacked effective processes to engage with African black community to assess, understand and attend their health care needs.
8	Kirby et al. (2018).	“It doesn’t exist. . .”: negotiating palliative care from a culturally and linguistically diverse patient and caregiver perspective.	To develop a critical, evidence-based understanding of the experiences of people from Culturally and Linguistically Diverse (CALD) backgrounds, and their caregivers, in a palliative care setting.	16 patients and 14 caregivers from a range of CALD backgrounds participated in semi-structured interviews.	Australia	Four themes were identified among participants: (1) Terminology in the transition to palliative care; (2) Communication, culture and pain management; (3) (Not) Talking about death and dying; and, (4) Religious faith as a coping strategy: challenging the terminal diagnosis.
9	Smith et al. (2009)	Palliative care for Latino patients and their families.	To understand Latinos palliative care needs and provide concrete suggestions.	Case study of 1 Latino woman diagnosed with leukeamia.	USA	Provided recommendations for issues identified related to culture, religious/spiritual, trustworthiness, health literacy and racial discrimination.
10	Eckemoff et al. (2018)	End of Life Care for Older Russian Immigrants - Perspectives of Russian Immigrants and Hospice Staff.	Examined immigrant Russian seniors and adult children’s views on end of life care and hospice staff members experiences providing care to diverse immigrant clients.	Qualitative research. 3 groups of participants. (1) 4 female Russian immigrant seniors aged 60 years and over, (2) 5 (2 male, 3 female) children of Russian seniors. (3) 4 female hospice staff.	North Carolina, USA	1. Preferred family carer. 2. Participants had different perception of end of life care. 3. Participants had different views about end of life care due to acculturation. 4. Lack of Advance Care Planning. 5. Participants felt that society should take some responsibility in caring for seniors.
11	Carlsson and Hjelm (2021).	Equal palliative care for foreign-born patients: a national quality register study.	To use data from a national quality register to investigate if there are differences relating to migrant background in the quality of end-of-life care of patients dying in Sweden.	81,418 deceased patients, over 18 year of age, registered in the Swedish Register of Palliative Care during 2017 and 2018, of expected death were included in the study.	Sweden	There were several significant differences in various quality indicators but not in a specific direction. Sometimes, the quality indicators showed an advantage for Swedish-born patients but just as often, they were also favorable for foreign-born patients. Swedish-born patients had greater access to specialized palliative care than foreign-born patients. Foreign-born patients were more often cared for in general home care setting, despite a higher frequency of cancer diagnosis.
